# Relationship between treatment-seeking behaviour and artemisinin drug quality in Ghana

**DOI:** 10.1186/1475-2875-11-110

**Published:** 2012-04-06

**Authors:** Eili Y Klein, Ian A Lewis, Christina Jung, Manuel Llinás, Simon A Levin

**Affiliations:** 1 Department of Ecology and Evolutionary Biology, Princeton University, Princeton, NJ, USA; 2 Lewis-Sigler Institute for Integrative Genomics, Princeton University, Princeton, NJ, USA; 3 Molecular Biology Department, Princeton University, Princeton, NJ, USA

**Keywords:** Anti-malarial drugs, Counterfeit, Artemisinin, Drug quality, Ghana

## Abstract

**Background:**

Artemisinin-based combination therapy (ACT) is currently the recommended first-line treatment for uncomplicated malaria infections. However, a significant proportion of ACT is assumed to be of poor quality, particularly in Africa. In addition, little is known about how treatment-seeking behaviour of individuals or drug price is associated with drug quality.

**Methods:**

Caregivers of children less than 5 years of age were interviewed on their knowledge of malaria and their choices for treatment. Artemisinin drugs were then purchased from sellers that caregivers preferred or had previously patronized. The active ingredients were quantified by nuclear magnetic resonance spectroscopy.

**Results:**

A negative relationship was anticipated between the education level of caregivers and the quality of anti-malarial drugs purchased. However, of the 33 drugs collected from 16 different shops, only one contained less than 80% of its purported active ingredient, and most drugs were within 90% of their listed amounts. No link was found between drug quality and price. Nonetheless, while ACT is the recommended first-line treatment in Ghana, 21% of the drugs collected were artemisinin monotherapy, and 27% of the ACT was not co-formulated. Among caregivers, higher education was found to be associated with both an increased likelihood of seeking treatment in a clinic first, as opposed to visiting drug shops or using herbal remedies, and with purchasing drugs from licensed sellers.

**Conclusion:**

Surprisingly, drug quality was found to be uniformly high and thus no significant relationship between price, treatment-seeking behaviour and the content of the active ingredients was observed. However, artemisinin monotherapy, which the WHO considers inappropriate therapy, was still widely available in Ghana in 2010. Monotherapy was more likely to be available in unlicensed vendors where less-educated caregivers generally shopped. This linkage between education, treatment-seeking behaviour and drug availability suggests that the global subsidy to reduce the cost of co-formulated ACT can play a significant role in increasing its availability.

## Background

It is estimated that 350 to 500 million Africans fall sick with malaria each year [1]; many of them have poor access to public health facilities and rely primarily on self-medication through the unregulated private and informal drug sector [2]. A large portion of anti-malarial medicines sold in Africa are believed to be counterfeit (fraudulently mislabelled) or substandard (products with 80% or less of the listed active ingredient) [3-5]. Whether drug vendors sell substandard medicines intentionally or unknowingly, low quality drugs increase morbidity and mortality by failing to properly treat malaria and contribute to the spread of anti-malarial drug resistance [4].

Artemisinin-based combination therapy (ACT) has been the WHO-recommended option for first-line malaria treatment since 2001. ACT combines an artemisinin-based active ingredient with another, slower-clearing anti-malarial compound that reduces the likelihood of resistance emerging to artemisinin [6-8]. ACT can also act to slow the spread of resistance [9], an important issue as resistance to artemisinin has been recently observed in Cambodia [10]. Thus, widespread use of substandard ACT both endangers lives and accelerates the rate at which resistance to artemisinin spreads. Similar quality problems with the former first-line drugs sulphadoxine-pyrimethamine (SP) and chloroquine (CQ) are believed to have contributed significantly to the spread of resistance to those drugs [4].

Because artemisinin compounds are derived from the plant *Artemisia annua*, which takes a long time to grow and produces low yields [11], drug production remains expensive and inefficient [12]. Despite recent increases in production, arteminisin monotherapy generally cost ten times more than other drugs [13], and ACT costs even more than monotherapy. Consequently, sophisticated counterfeits have been reported in Asia [14], resulting in a number of cases of increased morbidity and mortality in the early part of the 21^st ^Century [15]. This generated concern that a similar problem would emerge in Africa [15,16].

Over the last ten years, numerous studies have measured drug quality in sub-Saharan Africa [3,17-24]. Although no consistent results have emerged from these studies, it is still widely believed that counterfeit and substandard artemisinin drugs are readily available across Africa [4,25].

Previous studies have not addressed how characteristics of individuals influence the quality of the drugs they purchase. For instance, evidence suggests that educational attainment by mothers, who are the main caregivers, is generally associated with better use of available health resources [26]. Thus, this study attempted to examine whether or not there was a relationship between education, treatment seeking behaviour by caregivers and the quality of the artemisinin drugs available at the places where they typically buy drugs following guidelines for field surveys of the quality of medicines [27]. Drug quality was assessed quantitatively using nuclear magnetic resonance (NMR) spectroscopy.

## Methods

### Study participants

Drug purchasing habits and the quality of ACT were studied in two peri-urban neighbourhoods (Osu and Osu Klottey) in Accra, Ghana. From July to August 2009 interviews were conducted with individuals living in Kokrobitey, a fishing village on the southern coast of Ghana about 19 miles west of Accra. All interviews were conducted in English about disease awareness and treatment-seeking behaviour (see guided questions in Additional file 1). Eligible participants were parents 15-50 years old with at least one child under five years of age and residing in Kokrobitey village at the time of study. Eligible participants were invited to participate during routine home visits. All caregivers that met requirements during visits on randomly selected days were invited to participate. There were no refusals. Informed and signed consent was obtained from each study participant according to the Institutional Review Board of Princeton University's confidentiality and consent guidelines (Protocol No. 4450).

### Drug sampling

Drugs were collected by a single non-native individual in January 2010 from all of the vendors mentioned by the study participants. Vendors were located in the Osu, Makola and Kaneshie Markets near the city of Accra. Establishments were categorized as licensed sellers if they had at least one licensed pharmacist or medical professional. Samples of artemisinin combination drugs were requested, but any artemisinin-related drug available was purchased. Samples of both combinations and monotherapy were acquired in a variety of formulations including tablets, powders, and syrups. Drug packaging was examined carefully for signs of tampering or counterfeiting.

### Preparation of drug samples

Three samples of each drug were homogenized and dissolved in either chloroform (artemether, dihydroartemisinin) or methanol (artesunate) over a 2-3 h period. The volume of solvent used for the extraction was adjusted to maintain a constant ratio between the solvent and the target drug (2 mg/ml for artemether drugs, 5 mg/ml for artesunate drugs, and 5 mg/ml for dihydroartemisinin). Samples were centrifuged for 10 min at 10,000 × g. An aliquot (1 ml) of each supernatant was transferred to a fresh tube and dried under a stream of N_2 _gas. Samples were re-dissolved in 1 ml of the corresponding perdeuterated solvent and 600 μL were transferred to 5 mm NMR tubes for quantitative analysis. Samples that showed lower than expected levels of drug were re-dissolved at twice the original dilution (2.5 mg/ml) and re-analysed by NMR. This step was included to ensure that solubility limits were not influencing our observed concentrations.

### NMR data analysis

NMR is a well-established tool for identifying and quantifying small molecules in complex mixture [28], and previous studies have used NMR to identify counterfeit drugs from field samples [29,30]. One dimensional ^1^H NMR spectra were collected on a Bruker Advance 500 MHz spectrometer equipped with a triple resonance cryoprobe. Spectra were acquired in four transients with four steady-state transients, an acquisition time of 4 s and initial delay of 11 s. The long recycle delay employed in this study minimizes quantitative artifacts related to differential *T1 *relaxation between resonances. Data were Fourier transformed, zero filled, phased, and drift corrected using Mnova software. All subsequent data analyses were performed using the open-source rNMR software package [31]. Drug resonances were identified and quantified using established methods [28]. Briefly, standards of each drug were prepared at multiple concentrations (e.g. 2, 5, 10, and 50 mM; N = 3 per concentration) and two or more non-overlapped resonances were identified for each compound (Additional file 2). The slope of the concentration *versus *intensity curve was then determined for each resonance by linear regression (Additional file 3). This regression statistic was then used to calculate concentrations of drugs observed in the test samples. For artemether-lumefantrine samples, standards were prepared from the reference drug samples for artemether (Sigma) and lumefantrine (Sigma) as well as from a Coartem^® ^reference standard provided by Novartis. Reported results are for the Coartem^® ^reference standard. All other drugs are reported relative to purchased drug standards (Sigma). All NMR data were collected at the NMR facility at Princeton University.

### IC_50 _studies

To cross validate the analytical results with a biological assay, drug concentrations observed in the NMR assay were compared to IC_50 _values derived from the same samples. *Plasmodium falciparum *cultures (3D7) were maintained using established methods [29]. Briefly, parasites were cultured in washed human erythrocytes incubated in RPMI 1640 medium (Sigma) supplemented with Albumax (Invitrogen, 2.5 g/L), HEPES (25 mM), hypoxanthine (100 μM), NaHCO3 (24 mM), and gentamycin (50 ng/L). Cultures were synchronized using sorbitol (5% sorbitol in PBS for 5 min at 37°C) and grown until they reached 2% parasitaemia. Drugs were serially diluted into 96-well plates (440 nM to 13 pM) and ring-stage parasites were aliquoted into each well (0.5% parasitaemia and 2% HCT, final). Plates were incubated for 72 h at 37°C, then frozen for 48 h at -20°C. Plates were then thawed for 3 h at room temperature and a 100 μl of culture was transferred to optical plates containing 100 μl of SYBR green I (Sigma) in lysis buffer (20 mM Tris-HCl; 5 mM EDTA; 0.08% TritonX-100; 0.008% saponin in PBS). Fluorescence was measured using a BioTek plate reader with excitation and emission wavelengths of 485 nm and 535 nm, respectively. IC_50 _values for each drug were calculated using ICEstimator [32].

### Role of the funding source

The institutions that supported this work had no role in study conception, data collection, analysis and interpretation, writing of the manuscript, or decision to submit for publication. All authors had full access to all data in the study.

## Results

### Field interviews

From July to August 2009, interviews were conducted in 34 households near Accra, Ghana. Both parents were interviewed in two households, while only the mother was interviewed in 32 households. Study participants were 20-43 years old, and all but two were married. All parents were the primary caregivers for at least one child under five. Table 1 lists the background profile information such as the age, education, household size and occupation of the parents who participated in the study (see Additional file 4 for full data on all caregivers).

**Table 1:** Profile of study participants

Characteristics	N (%)
**Age**	
15-24	8 (22%)
25-34	24 (67%)
35-44	4 (11%)
**Educational Status**	
No formal education	2 (6%)
Primary (1-6)	28 (78%)
Secondary (7-12)	6 (17%)
**Total living children living in household**	
1	5 (14%)
2	11 (31%)
3	8 (22%)
4	7 (19%)
5	3 (8%)
6	2 (6%)
**Living children under five in household**	
1	15 (42%)
2	15 (42%)
3	6 (17%)
**Occupation**	
farmer	11 (31%)
market vendor	21 (58%)
housewife	2 (6%)
student	1 (3%)
unemployed	1 (3%)

All parents recognized malaria as a widespread disease transmitted through mosquitoes, and engaged in some form of preventive behaviour, such as burning mosquito coils and draining areas of still water near the house. All study participants had previously treated themselves and/or their children for malaria. All parents reported previously having malaria, and 25 (69%) parents reported having dealt with malaria in their children. Thirty three (92%) parents reported fever, shivering and chills, and headache as the predominant symptoms of malaria they looked for in their children.

The most popular first, second and third preferences for treatment were market drug vendors, herbal remedies, and public health facilities, respectively (Table 2). While all but one caregiver (97%) listed the drug shop/medicine seller as one of their top three choices for seeking treatment, nearly as many (86%) listed traditional/herbal remedies. Level of education influenced the preferred source of treatment and perceived quality at medicine sellers that parents most frequently visited. Five of the six parents with a secondary education or higher listed a public or private health facility as their first choice for seeking malaria treatment, and five of six also mentioned licensed shops when asked to give examples of their preferred medicine sellers (the last one did not list a preference). In addition, these parents were more likely not to list traditional/herbal remedies at all.

**Table 2:** Reported priority of malaria treatment sources

Source of treatment	First choice	Second choice	Third choice
Drug shop or market medicine vendor	17 (47%)	11 (31%)	7 (19%)
Traditional and herbal remedies	9 (25%)	16 (44%)	6 (17%)
Public health facility	6 (17%)	6 (17%)	17 (47%)
Private clinic	2 (6%)	3 (8%)	6 (17%)
Other (leftover/borrowed medicines)	2 (6%)	0 (0%)	0 (0%)

### Drug quality

In total, 33 artemisinin-based malarial medicines were purchased from licensed urban pharmacies and informal market vendors cited as anti-malarial drug sources by at least one parent (Additional file 5). Nine of the samples were purchased from five licensed sellers in Osu and 24 from 11 informal sellers in Makola and Kaneshie Markets. Fifteen of the drugs were artemether-based drugs, 12 of the drugs were artesunate drugs and the remaining six were dihydroartemisinin drugs. At two of the informal sellers, ACT was not available, so artemisinin monotherapies were purchased instead. In addition, artemisinin monotherapies were readily available and purchased at four other vendors including one licensed seller.

The drugs collected were produced by 13 different manufacturers. The most common was Bliss GVS, an Indian company, which accounted for almost half of all the drugs purchased, followed by companies from China and Ghana. All the drugs were purchased in January 2010, and all but two had a remaining shelf life of at least six months. Of the ones with a shelf life less than six months, both were purchased separately from two unlicensed drug vendors in Makola Market and had listed expiration dates of Nov 2009 and May 2010. Despite the expiration date, all drugs were included in the final analysis.

All the artemether-based drugs collected were available in combination with lumafantrine; 10 were co-formulated combination tablets, three were dry powder formulations, and two had separate tablets for artemether and lumefantrine. Eight of the 12 artesunate-based drugs were available as an ACT, however only three of them were co-formulated. Of the six dihydroartemisinin samples, three were monotherapy, two were co-formulated with piperaquine (one of which was a powder) and the final sample was a tri-formulated tablet with piperaquine and trimethoprim.

**Figure 1:**
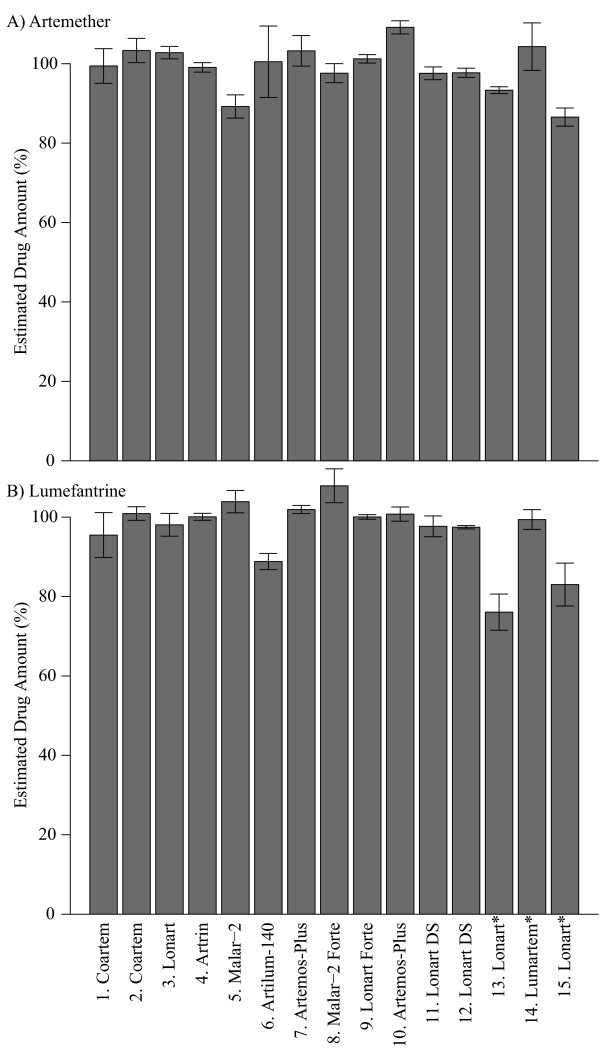
**Artemether-lumefantrine**. Percentage of each drug found relative to the stated quantity on the box as measured against Coartem^® ^standard (Novartis). * powder formulations for which the extraction method was not optimized.

Drugs were quantified using established NMR methods [28,31]. Only one of the artemether-based drugs, a powder formulation, was found to be below 80% of the expected amount (Figure 1 and Additional file 6 and Additional file 7). However, powders can be problematic because they can absorb water and thereby artificially deflate yields. In addition, the relatively large amount of excipients present in powder formulations can affect drug solubility and can create other solution chemistry problems that interfere with quantitative analysis. Thus, the low yield observed in this sample is inconclusive and may be due to absorption of water or solution chemistry problems rather than a reflection that this drug is of substandard quality.

**Figure 2:**
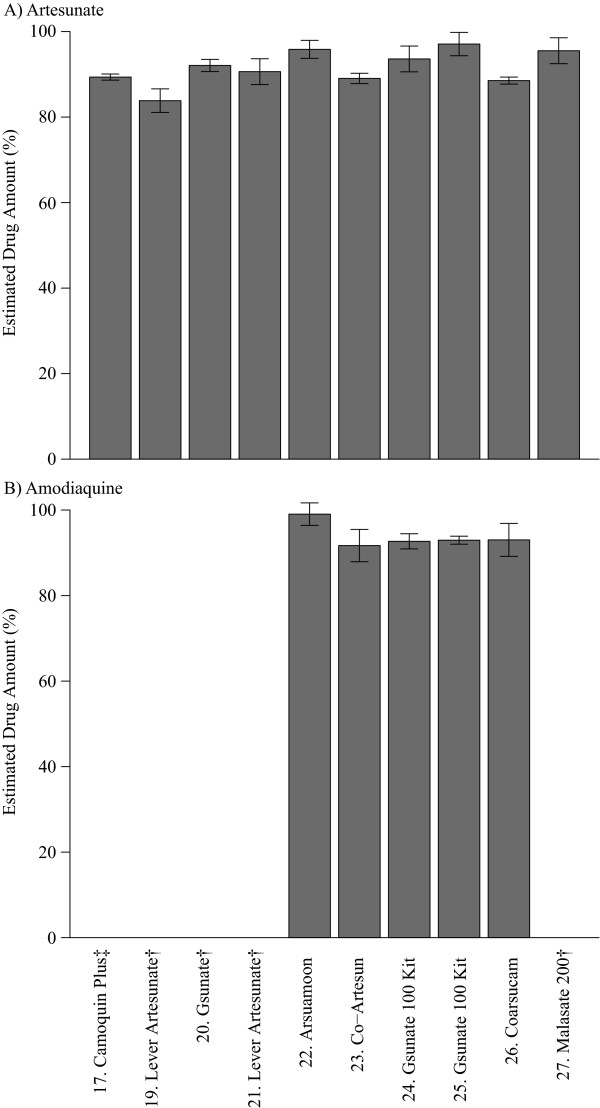
**Artesunate-amodiaquine**. Percentage of each drug found relative to the stated quantity on the box as measured against a reference standard (Sigma). † artesunate monotherapy drugs; ‡ amodiaquine was a liquid suspension that was not optimized for extraction method. Number 16 was a suppository for which extraction was not optimized and number 18 was opened prior to analysis.

**Figure 3:**
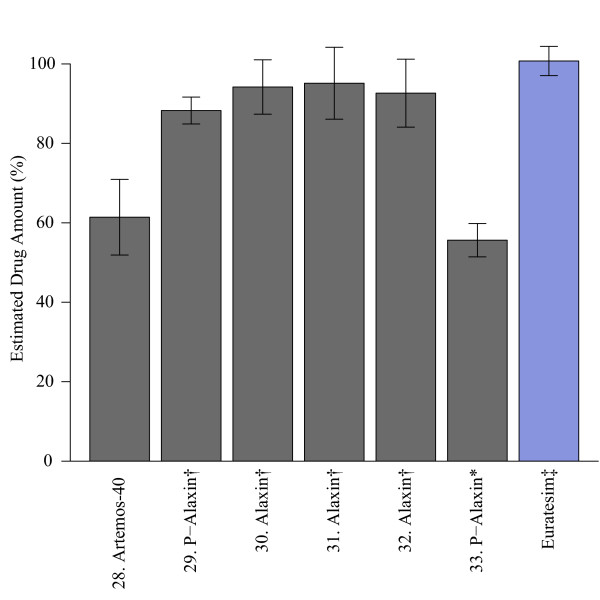
**Dihydroartemisinin**. Percentage of each drug found relative to the stated quantity on the box as measured against a reference standard (Sigma). * powder formulations for which the extraction method was not optimized; † dihydroartemisinin monotherapy drug; ‡ co-formulated drug standard (Sigma-Tau).

For artesunate-based formulations, observed drug levels consistently matched the content stated on the package (Figure 2 and Additional file 8 and Additional file 9). Two non-tablet drugs, #16 (co-formulated suppository) and #17 (amodiaquine suspension only) were excluded because the extraction method was not optimized for these drugs. Number 18 was opened prior to testing so was also excluded from the analysis. For dihydroartemisinin-based formulations, two drugs were below their expected levels (Figure 3); one sample (#28) was a tri-formulated drug and the other was a powder mixture (#33). Observed levels of both drugs were consistent across multiple dilutions (Additional file 10), indicating that solubility limits were not influencing yields. As mentioned previously, powder formulations are subject to quantitative problems, making it difficult to determine whether the drug was of substandard quality. However, based on these results it can be concluded that the level of dihydroartemisinin in the tri-formulated drug was less than the amount stated on the package.

To assess the efficacy of NMR as a quantitative tool, the observed concentrations prior to dilution of the dihydroartemsinin drugs were cross-validated with a biological activity assay. IC_50 _values of the sample drugs relative to both a dihydroartemisin standard and a co-formulated drug standard, Eurartesim™, were measured. The resulting IC_50 _values showed significant correlation between the observed concentration and biological activity (Additional file 11).

## Discussion

The widespread use of the highly effective anti-malarial drug, CQ, led to the emergence of resistance and the eventual demise of the first malaria eradication effort. During the 1980s, circulation of substandard CQ anti-malarial medicines in Africa contributed to the subsequent spread of CQ resistance in parts of eastern Africa [4,20]. Sulphadoxine-pyrimethamine, the first-line drug widely adopted after CQ resistance emerged, suffered a similar fate in the 1990s [4]. The recent introduction of widespread artemisinin use has revived talk of malaria eradication [33]. However, sub-therapeutic use of this drug due to counterfeiting may imperil its efficacy and spread resistance faster. This is particularly true of drug purchases from unlicensed sellers and the informal drug market, which is the primary source of pharmaceutical products for patients or caregivers seeking home-based malaria treatment in many rural areas where the formal health system does not reach [34]. In this study, the link between consumer education, treatment-seeking behaviour and the quality of the drugs purchased was examined.

The results from field interviews suggest that increased education was associated with an increased likelihood to patronize licensed drug sellers and to rely less heavily on traditional remedies. This is in accord with research showing increased education of mothers is associated with a higher use of western health services [26]. Increased education is also often associated with increased wealth; thus it was not surprising that there was a moderate cost difference between shops that had licensed sellers and those that did not. At licensed shops, the average cost of drugs was 6.63 cedis (US$ 4.65) compared to 5.07 cedis (US$ 3.55) at non-licensed shops. This effect was apparent even if the monotherapy drugs, which were more likely to be sold at non-licensed shops and which were on average just over half the cost of the ACT (6.05 cedis *vs *3.44 cedis or US$ 4.24 *vs *US$ 2.41), were excluded.

Based on prior reports [3,14,17-19,24], the expectation was that a large percentage of the drugs would be of low quality. However, consistently high drug quality across the samples collected for this study was observed. Only three drugs fell below 80% of the expected level, the pass/fail threshold used by other studies [3,18]. Two of these drugs were powder formulations, which are subject to a variety of analytical problems that prevent us from drawing any definitive conclusions about their quality. However, the third drug was a tablet formulation that showed 60% of the expected drug quantity across multiple dilutions. From these data, it can be concluded that the amount of dihydroartemisinin contained in sample 28 was less than the amount stated on the package.

Despite high overall drug quality, the number of artemisinin monotherapy drugs available for purchase suggests a deeper structural problem in the drug market. While ACT is the recommended first-line treatment in Ghana, and the WHO has called for an end to the production and marketing of artemisinin monotherapy, seven (21%) of the drugs purchased were artemisinin monotherapy, one of which was purchased at a licensed seller. Co-formulation was also a problem; seven forms (27%) of ACT were not co-formulated. Where drugs are not co-formulated, patients are likely to inadvertently or even knowingly use the drugs as monotherapy [6].

Despite vast cost differences, no linkage was found between the price and the quality of the drug. This result may be due to the limited number of samples collected or the fact that the drugs were purchased by a non-native person. However, this result is consistent with a large multi-country study, which found that price offered only a weak signal of drug quality [35]. Also, no real difference between the quality of drugs sold at licensed sellers *versus *unlicensed sellers was found, though only the quality of artemisinin-based drugs were sampled, and these are significantly more expensive than other drugs, and have been the focus of control efforts in Ghana [18].

In addition to the small sample size and the fact that the drugs were collected by a non-Ghanaian, no dissolution analysis was performed, samples were not blinded before analysis, and while packaging was examined carefully, no genuine examples were available to compare against. Another limitation of this study is that the bioavailability and efficacy of drugs can be influenced by excipients [36], which were not tested in this study. This limitation was partially addressed by comparing NMR-observed levels of the active ingredients to biological activity in an IC-50 assay. These data show that all of the formulations tested were effective *in vitro *despite their diverse excipients.

While no link was found between price and quality, it should be noted that the absolute price levels in 2010 were extremely high, averaging about 5.5 Ghanaian cedis (US$ 3.85), with a minimum of 2.8 cedis (US$ 1.96) and a maximum of 12 cedis (US$ 8.41). These rates are well above the cost that most ordinary Ghanaians can afford - the gross-national income per capita is estimated to be US$ 1,327 (Ghana Statistical Services). This suggests that the Affordable Medicines Facility - malaria (AMFm) may be able to play a critical role in places such as Ghana. Designed as a global subsidy to reduce the cost of artemisinin co-formulated products, AMFm is intended to both reduce the use of monotherapy and substandard drugs, as well as expand access to ACT [6,37]. All of the drugs in this study were collected prior to the implementation of the subsidy. Thus, this report can serve as a benchmark in examining the impact of the AMFm subsidy both for quality of ACT and the availability of monotherapy, which is a significant problem for containing the spread of artemisinin resistance that has recently emerged in Cambodia [10].

## Conclusions

The results from this study suggest that the quality of drugs available for purchase in some parts of Ghana in 2010 was surprisingly high. These findings also indicate that recent high-profile reports [3,4,18] claiming low overall drug quality in Africa should not be generalized; some communities, such as the peri-urban neighbourhoods, Osu and Osu Klottey, in Accra, Ghana are receiving quality products. One distinction between this study and those reporting significant quality problems is the use of NMR. Although NMR is not applicable to field studies, it is a highly quantitative tool [38,39] that is routinely used for drug quality analyses [40,41]. Future field studies should cross-validate colorimetric assays [3,17,18] with established quantitative tools such as NMR or high-performance liquid chromatography mass spectrometry.

While the sample size of this study was small, the sampling method purposely sought places that may provide lower quality drugs, yet there was no apparent link between treatment-seeking behaviour and the quality of drugs purchased. Individuals with more education are more likely to seek treatment at a clinic and purchase drugs from licensed vendors, but the content of the active ingredients do not seem to vary significantly. However, the high cost of the drugs presents a significant access barrier. In addition, from the point of view of the long-term efficacy of the artemisinin, the continued availability of artemisinin monotherapy remains a significant problem. As the scale of artemisinin production continues to increase, it is important that countries remain vigilant about the possible problems of counterfeiting, but it is also important that the use of co-formulated artemisinin combination therapy is expanded to save lives and reduce the rate at which resistance to artemisinin spreads.

## Competing interests

The authors declare that they have no competing interests.

## Authors' contributions

EK and CJ developed the study design. CJ purchased the drugs, interviewed caregivers, and analysed data. EK and IL performed drug analysis. EK, IL and CJ wrote the manuscript. SL and ML reviewed and commented on the manuscript and contributed to the interpretation of the results. All authors saw and approved the final version.

## Supplementary Material

Additional file 1 **Guided Questions for Household Interviews**. Guided questions used to conduct all interviews with participants about disease awareness and treatment-seeking behaviour. Click here for file

Additional file 2 **Example comparing NMR Spectra of different compounds**. One-dimensional ^1^H NMR spectra of artemether standard, lumefantrine standard, and Coartem^®^, a coformulated standard, at different concentrations. Click here for file

Additional file 3 **Example comparing NMR Spectra of different concentrations**. One-dimensional ^1^H NMR spectra of artemether standards at different concentrations. Click here for file

Additional file 4 **Caregiver Information**. Table listing detailed information about each participating caregiver. Click here for file

Additional file 5 **Drug Formulations**. Detailed data on each drug collected. Click here for file

Additional file 6 **Artemether Estimated Quantity**. Raw data of estimated artemether concentrations. Click here for file

Additional file 7 **Lumefantrine Estimated Quantity**. Raw data of estimated lumefantrine concentrations. Click here for file

Additional file 8 **Artesunate Estimated Quantity**. Raw data of estimated artesunate concentrations. Click here for file

Additional file 9 **Amodiaquine Estimated Quantity**. Raw data of estimated amodiaquine concentrations. Click here for file

Additional file 10 **Dihydroartemisinin Estimated Quantity**. Raw data of estimated dihydroartemisinin concentrations. Click here for file

Additional file 11 **IC_50 _*vs*. Observed Drug Quantity**. Plot of estimated IC_50 _values *vs *observed drug quantity. Click here for file
