# Supplementary material for: Relationship between treatment-seeking behaviour and artemisinin drug quality in Ghana
Source: Malar J. 2012 Apr 6;11:110. doi: 10.1186/1475-2875-11-110 (PMC3339389; doi:10.1186/1475-2875-11-110)
Supplement: Additional file 1 — Guided Questions for Household Interviews. Guided questions used to conduct all interviews with participants about disease awareness and treatment-seeking behaviour. [file 1475-2875-11-110-S1.PDF]

Additional File 1: Guided Questions for Household Interviews
(July-August 2009)

1. What is your age?
2. What is your level of education?
3. How many members live in your household?
    a. How many children do you have?
    b. How old?
4. How much time, on average, do you spend at home in a week? Are you employed outside of the home?
5. Have you, or a member of your household, had malaria in the past? Previously sought professional or non-professional medical attention?

Malaria Awareness and Health Services

6. What causes malaria and how is it spread?
7. What are the symptoms of malaria? And what are those of cerebral malaria?
8. How can malaria be prevented and how can it be treated?
9. Where would you most likely seek advice, information, or help about health? (distance and cost?)

Treatment-Seeking Behavior

10. Has your child been ill with fever/convulsions at any time in the last year? [If yes, continue as written. If no, ask the following questions as hypothetical.)
11. Did you seek advice or treatment for the fever/convulsions? First, second, and third choices?
12. Was the child given any medicines for the fever/convulsions? If yes, what kinds, when, and for how long?
13. What other actions did you take, besides administer medicines?
