# Supplementary material for: Relationship between treatment-seeking behaviour and artemisinin drug quality in Ghana
Source: Malar J. 2012 Apr 6;11:110. doi: 10.1186/1475-2875-11-110 (PMC3339389; doi:10.1186/1475-2875-11-110)
Supplement: Additional file 2 — Example comparing NMR Spectra of different compounds. One-dimensional 1H NMR spectra of artemether standard, lumefantrine standard, and Coartem®, a coformulated standard, at different concentrations. [file 1475-2875-11-110-S2.PDF]

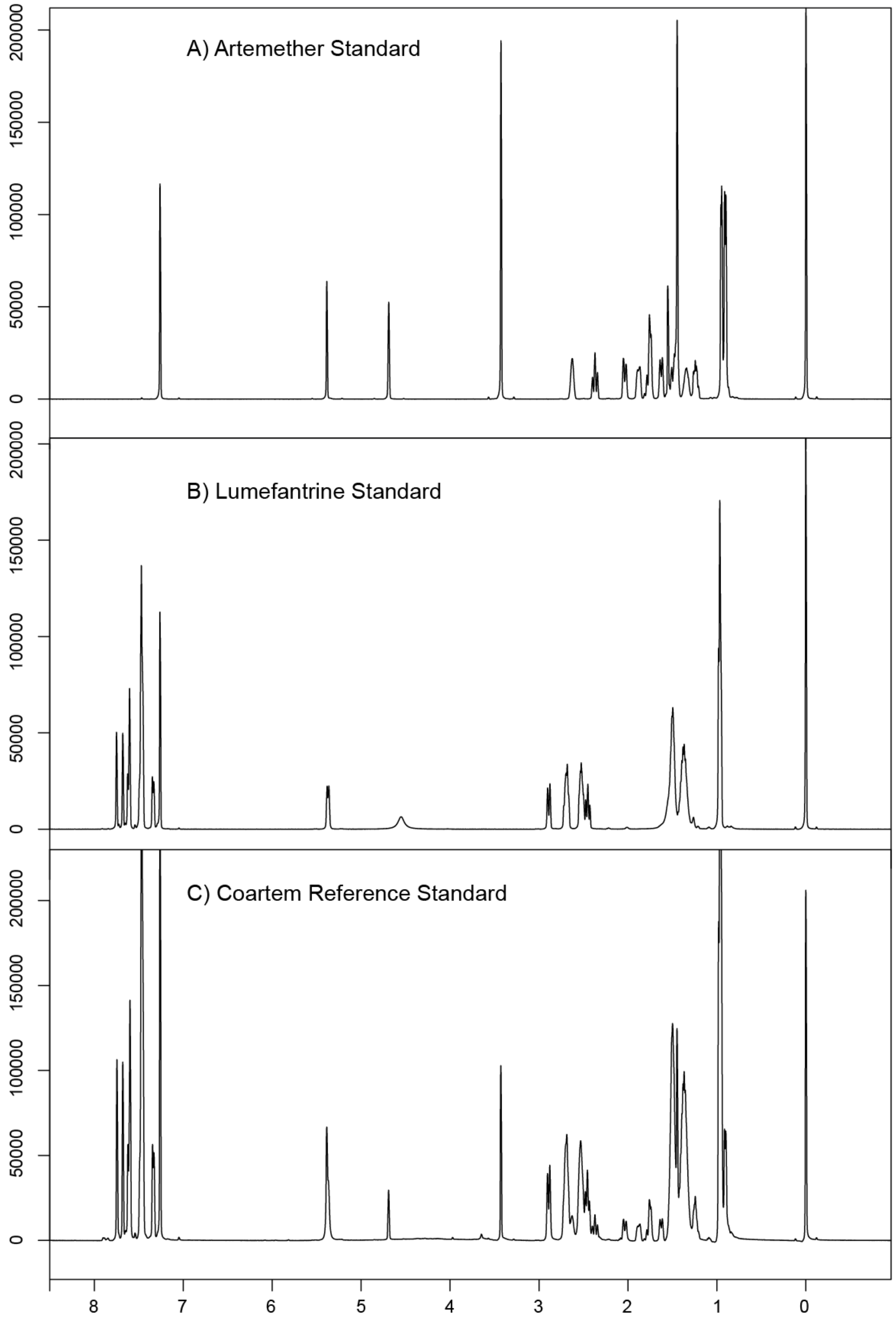

Additional File 2: One-dimensional ^1H NMR spectrum of A) an artemether standard, B) a lumefantrine standard, and C) Coartem, a coformulated ACT, containing both artemether and lumefantrine. The different spectra demonstrate the unique peaks that occur for each compound, and the fact that these are identifiable even when in combination. Artemether-specific peaks are visible in the range around 2ppm and between 3ppm and 4ppm, while lumefantrine-specific peaks are visible in the range between 7ppm and 8ppm.
