# Supplementary material for: Relationship between treatment-seeking behaviour and artemisinin drug quality in Ghana
Source: Malar J. 2012 Apr 6;11:110. doi: 10.1186/1475-2875-11-110 (PMC3339389; doi:10.1186/1475-2875-11-110)
Supplement: Additional file 3 — Example comparing NMR Spectra of different concentrations. One-dimensional 1H NMR spectra of artemether standards at different concentrations. [file 1475-2875-11-110-S3.PDF]

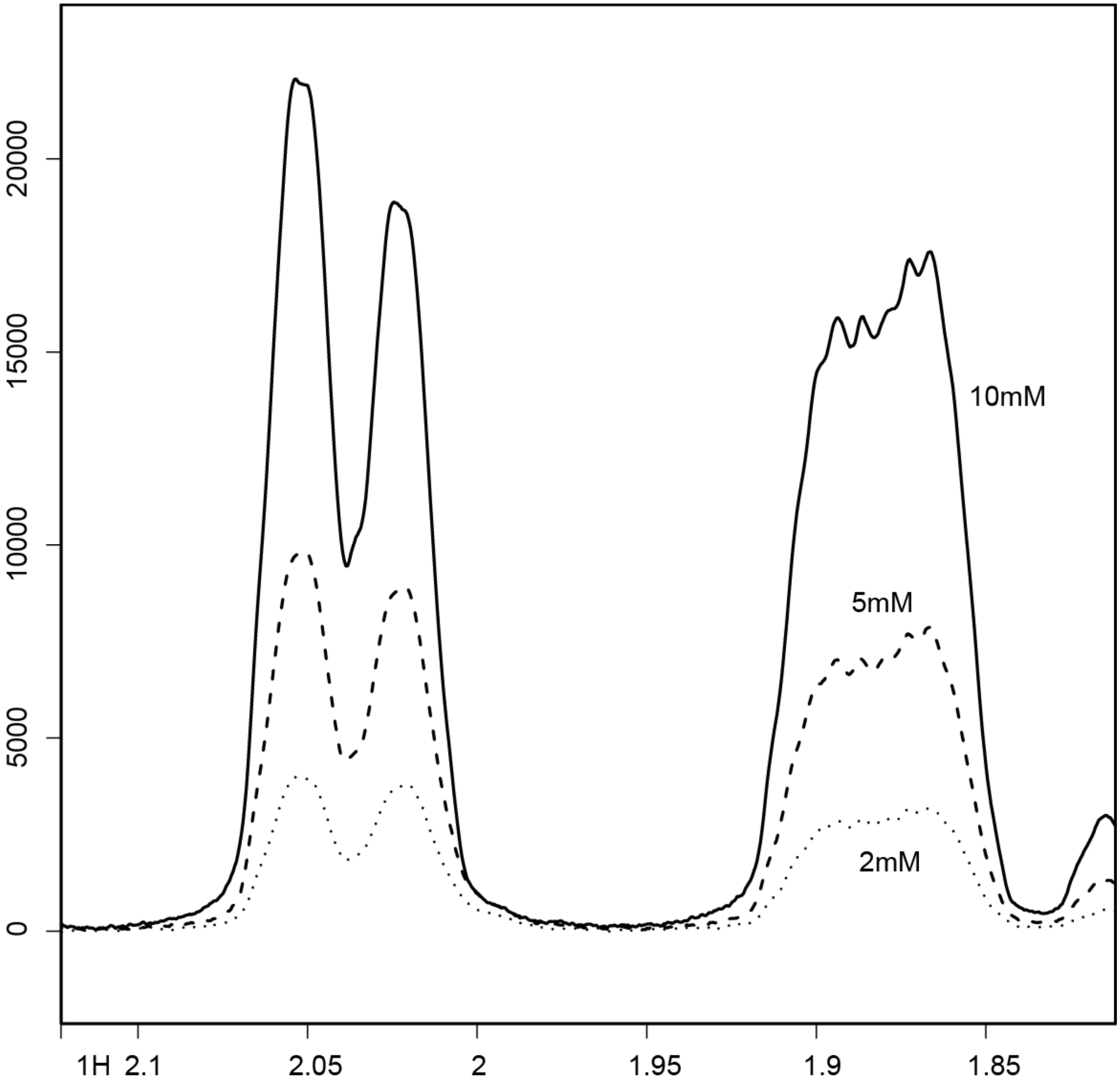

Additional File 3: One-dimensional ^{1}H NMR spectra of artemether standards at different concentrations. The empirically-determined peak intensity versus concentration function was used measure drug levels of samples.
