# Supplementary material for: Relationship between treatment-seeking behaviour and artemisinin drug quality in Ghana
Source: Malar J. 2012 Apr 6;11:110. doi: 10.1186/1475-2875-11-110 (PMC3339389; doi:10.1186/1475-2875-11-110)
Supplement: Additional file 4 — Caregiver Information. Table listing detailed information about each participating caregiver. [file 1475-2875-11-110-S4.PDF]

Additional File 4: Caregiver Information

Parent	Age	Education	Occupation	Living Children <5	Total living children in household	Treatment Option: 1st choice	2nd choice	3rd choice	Recommended Drug Shop
Mother	23	Primary	Farmer	1	1	Drug Shop	Public Health Facility	Traditional	Dove, Sadasko
Mother	20	Primary	Unemployed	1	1	Traditional	Drug Shop	Public Health Facility	None
Mother	22	Secondary/Above	Market Vendor	1	1	Private Clinic	Public Health Facility	Drug Shop	John Lawrence, Dove
Mother	24	Secondary/Above	Market Vendor	1	1	Drug Shop	Public Health Facility	Traditional	Pillbox
Mother	25	Secondary/Above	Student	1	1	Public Health Facility	Drug Shop	Private Clinic	Tropic, Richcord
Mother	30	Primary	Farmer	1	2	Traditional	Public Health Facility	Drug Shop	None
Mother	29	Primary	Market Vendor	1	2	Drug Shop	Traditional	Public Health Facility	Sarkuff, Dove
Mother	31	Secondary/Above	Market Vendor	1	2	Public Health Facility	Traditional	Drug Shop	John Lawrence
Mother	24	Secondary/Above	Market Vendor	1	2	Public Health Facility	Private Clinic	Drug Shop	Tropic
Mother	25	Primary	Housewife	2	2	Private Clinic	Public Health Facility	Drug Shop	Adler
Mother	23	Primary	Market Vendor	2	2	Drug Shop	Traditional	Public Health Facility	Bendoz

Mother	23	Primary	Market Vendor	2	2	Drug Shop	Traditional	Private Clinic	F&F, Gladon
Mother	27	Primary	Market Vendor	2	2	Drug Shop	Traditional	Private Clinic	None
Mother	26	Primary	Market Vendor	2	2	Drug Shop	Traditional	Private Clinic	K. Somuah & Sons
Mother	26	Primary	Market Vendor	2	2	Drug Shop	Traditional	Private Clinic	Adler, F&F
Mother	28	Secondary/Above	Market Vendor	2	2	Public Health Facility	Private Clinic	Traditional	None
Mother	34	Primary	Farmer	1	3	Traditional	Drug Shop	Public Health Facility	Adler
Mother	30	Primary	Market Vendor	1	3	Traditional	Drug Shop	Public Health Facility	None
Mother	23	Primary	Market Vendor	2	3	Public Health Facility	Private Clinic	Drug Shop	Josdav
Mother	25	Primary	Market Vendor	2	3	Public Health Facility	Drug Shop	Traditional	K. Somuah, Bendoz
Mother	28	Primary	Market Vendor	2	3	Traditional	Drug Shop	Public Health Facility	Bendoz
Mother	27	Primary	Farmer	2	3	Drug Shop	Traditional	Public Health Facility	Sadasko
Mother	27	Primary	Farmer	2	3	Drug Shop	Traditional	Public Health Facility	None
Mother	26	No Formal Education	Farmer	3	3	Other	Drug Shop	Traditional	None
Mother	32	Primary	Market Vendor	2	4	Drug Shop	Public Health Facility	Traditional	Aseda, GA Boateng
Mother	33	Primary	Market Vendor	2	4	Traditional	Drug Shop	Public Health Facility	None

Mother	26	Primary	Farmer	2	4	Drug Shop	Traditional	Public Health Facility	None
Mother	28	Primary	Market Vendor	3	4	Traditional	Drug Shop	Public Health Facility	Josdav
Mother	29	Primary	Market Vendor	3	4	Traditional	Drug Shop	Public Health Facility	none
Mother	28	Primary	Market Vendor	3	4	Traditional	Drug Shop	Public Health Facility	none
Mother	28	Primary	Market Vendor	3	4	Drug Shop	Traditional	Private Clinic	Adler
Mother	29	No Formal Education	Farmer	3	5	Other	Traditional	Drug Shop	Dove
Father	42	Primary	Farmer	1	5	Drug Shop	Traditional	Public Health Facility	Dove
Mother	39	Primary	Housewife	1	5	Drug Shop	Traditional	Public Health Facility	Dove
Father	43	Primary	Farmer	1	6	Drug Shop	Traditional	Public Health Facility	Dove, F&F
Mother	38	Primary	Farmer	1	6	Drug Shop	Traditional	Public Health Facility	Dove, F&F
