# Supplementary material for: Relationship between treatment-seeking behaviour and artemisinin drug quality in Ghana
Source: Malar J. 2012 Apr 6;11:110. doi: 10.1186/1475-2875-11-110 (PMC3339389; doi:10.1186/1475-2875-11-110)
Supplement: Additional file 5 — Drug Formulations. Detailed data on each drug collected. [file 1475-2875-11-110-S5.PDF]

Additional File 5: Drug Formulations

Drug Name	Listed Manufacturer (location)	Coformulate(s)	Dosage Form (Amt)	Claimed Strength§	Cost (Ghc)	Source
**Artemether**						
1. Coartem	Novartis (Beijing, China)	Lumefantrine	Tablet (24)*	20/120	11	Pillbox**
2. Coartem	Novartis (Beijing, China)	Lumefantrine	Tablet (24)*	20/120	12	Gladon
3. Lonart	Bliss GVS (Mumbai, India)	Lumefantrine	Tablet (24)*	20/120	4	Gladon
4. Artrin	Medreich plc (Surrey, England)	Lumefantrine	Tablet (24)*	20/120	7.6	Primrose**
5. Malar-2	Ernest Chemists Ltd. (Accra, Ghana)	Lumefantrine	Tablet (24)*	20/120	6.5	F&F
6. Artilum-140	Ronak Exim PVT. Ltd. (Gendigate, India)	Lumefantrine	Tablet (24)*	20/120	4.5	Adler
7. Artemos-Plus	ETDZS Industry Ltd. (Chongqing, China)	Lumefantrine	Tablet (12 of each)	40/240	5.5	F&F
8. Malar-2 Forte	Ernest Chemists Ltd. (Accra, Ghana)	Lumefantrine	Tablet (12)*	40/240	5.5	Sadasko
9. Lonart Forte	Bliss GVS (Mumbai, India)	Lumefantrine	Tablet (12)*	40/240	4	Gladon
10. Artemos-Plus	ETDZS Industry Ltd. (Chongqing, China)	Lumefantrine	Tablet (12 of each)	40/240	6	Tropic**
11. Lonart DS	Bliss GVS (Maharashtra, India)	Lumefantrine	Tablet (6)*	80/480	6	Richcord**
12. Lonart DS	Bliss GVS (Mumbai, India)	Lumefantrine	Tablet (6)*	80/480	5	Gladon
13. Lonart	Bliss GVS (Maharashtra, India)	Lumefantrine	Dry Powder (24g)*	180/1080	5.5	K. Somuah & Sons
14. Lumartem	Cipla, Ltd. (India)	Lumefantrine	Dry Powder (60ml)*	180/1080	3.5	Sarkuff
15. Lonart	Bliss GVS (Maharashtra, India)	Lumefantrine	Dry Powder (24g)*	180/1080	6	Josdav Chemists Ltd.
**Artesunate**						
16. Gsunate Plus 25	Bliss GVS (Maharashtra, India)	Amodiaquine	Suppositories (6)*	25/75	6.2	John Lawrence Chemists Ltd. **
17. Camoquin Plus	Pfizer Afrique de l'Ouest (Dakar, Senegal)	Amodiaquine	Dry Powder (6 packets) and Camoquin suspension	25/50	7	Sadasko
18. Camosunate Ped	Danadams Pharma Industry Ltd. (Accra, Ghana)	Amodiaquine	Dry Powder packets (3 of each)	50/150	5.5	Sadasko
19. Lever Artesunate	Adams Pharmaceutical Co. (Anhui, China)		Tablet (12)†	50	3.5	Dove
20. Gsunate	Bliss GVS (Maharashtra, India)		Tablet (12)†	50	3	Bendoz
21. Lever Artesunate	Adams Pharmaceutical Co. (Anhui, China)		Tablet (12)†	50	4.7	GA Boateng
22. Arsuamoon	Guilin Pharmaceutical (Guilin, China)	Amodiaquine	Tablet (12 of each)	50/150	3.9	John Lawrence Chemists Ltd. **
23. Co-Artesun	Guilin Pharmaceutical (Guilin, China)	Amodiaquine	Tablet (12)*	50/153	6.5	K. Somuah & Sons
24. Gsunate 100 Kit	Bliss GVS (Mumbai, India)	Amodiaquine	Tablet (6 of each)	100/300	4	Adler

25. Gsunate 100 Kit	Bliss GVS (Mumbai, India)	Amodiaquine	Tablet (6 of each)	100/300	3.8	Dove
26. Coarsucam	Sanofi Aventis (Casablanca, Morocco)	Amodiaquine	Tablet (6)*	100/270	11.2	Tropic**
27. Malasate 200	Ernest Chemists Ltd. (Accra, Ghana)		Tablet (6)†	200	4.5	Sadasko
**Dihydroartemisinin**						
28. Artemos-40	ETDZS Industry Ltd. (Chongqing, China)	Piperaquine/ Trimethoprim	Tablet (8)*	32/320/90	5	Tropic**
29. P-Alaxin‡	GVS Labs (Dombivli, India)	Piperaquine	Tablet (8)*	40/320	4.5	Adler
30. Alaxin	Bliss GVS (Palghar, India)		Tablet (8)†	60	2.8	K. Somuah & Sons
31. Alaxin	Bliss GVS (Palghar, India)		Tablet (8)†	60	2.8	Tropic**
32. Alaxin	Bliss GVS (Palghar, India)		Tablet (8)†	60	2.8	GA Boateng
33. P-Alaxin	Bliss GVS (Mumbai, India)	Piperaquine	Dry Powder (80ml)*	80/640	7	Aseda

* co-formulated drug; † monotherapy; ‡ expired; ** licensed seller; § for co-formulated drugs, artemisinin derivative listed first
