# Supplementary material for: Relationship between treatment-seeking behaviour and artemisinin drug quality in Ghana
Source: Malar J. 2012 Apr 6;11:110. doi: 10.1186/1475-2875-11-110 (PMC3339389; doi:10.1186/1475-2875-11-110)
Supplement: Additional file 6 — Artemether Estimated Quantity. Raw data of estimated artemether concentrations. [file 1475-2875-11-110-S6.PDF]

Additional File 6: Artemether Estimated Quantity

Drug Name (Source)	Trial-1	Trial-2	Trial-3	Average	SD	Expected	Percent
1. Coartem (Pillbox)	6.74	6.34	6.91	6.66	0.29	6.70	99%
2. Coartem (Gladon)	7.15	6.75	6.86	6.92	0.21	6.70	103%
3. Lonart (Gladon)	6.98	6.91	6.77	6.89	0.11	6.70	103%
4. Artrin (Primrose)	6.55	6.68	6.68	6.64	0.08	6.70	99%
5. Malar-2 (F&F)	5.84	5.89	6.20	5.98	0.20	6.70	89%
6. Artilum-140 (Adler)	6.04	7.14	7.02	6.73	0.60	6.70	100%
7. Artemos-Plus (F&F)	6.87	6.69	7.20	6.92	0.26	6.70	103%
8. Malar-2 Forte (Sadasko)	6.52	6.71	6.39	6.54	0.16	6.70	98%
9. Lonart Forte (Gladon)	6.84	6.81	6.70	6.78	0.07	6.70	101%
10. Artemos-Plus (Tropic)		7.39	7.24	7.31	0.11	6.70	109%
11. Lonart DS (Richcord)	6.61	6.41	6.59	6.54	0.11	6.70	98%
12. Lonart DS (Gladon)	6.50	6.64	6.50	6.55	0.08	6.70	98%
13. Lonart* (K. Somuah & Sons)	6.32	6.20	6.24	6.25	0.06	6.70	93%
14. Lumartem* (Sarkuff)	7.12	7.31	6.54	6.99	0.40	6.70	104%
15. Lonart* (Josdav Chemists Ltd.)	5.79	5.65	5.96	5.80	0.15	6.70	87%

* Dry powder formula
