# Supplementary material for: Relationship between treatment-seeking behaviour and artemisinin drug quality in Ghana
Source: Malar J. 2012 Apr 6;11:110. doi: 10.1186/1475-2875-11-110 (PMC3339389; doi:10.1186/1475-2875-11-110)
Supplement: Additional file 7 — Lumefantrine Estimated Quantity. Raw data of estimated lumefantrine concentrations. [file 1475-2875-11-110-S7.PDF]

Additional File 7: Lumefantrine Estimated Quantity

Drug Name (Source)	Trial-1	Trial-2	Trial-3	Average	SD	Expected	Percent
1. Coartem (Pillbox)	21.28	20.58	23.07	21.64	1.28	22.67	95%
2. Coartem (Gladon)	23.19	22.44	22.99	22.87	0.39	22.67	101%
3. Lonart (Gladon)	22.47	22.72	21.50	22.23	0.65	22.67	98%
4. Artrin (Primrose)	22.53	22.91	22.63	22.69	0.20	22.67	100%
5. Malar-2 (F&F)	24.13	23.66	22.87	23.55	0.64	22.67	104%
6. Artilum-140 (Adler)	19.63	20.23	20.54	20.14	0.46	22.67	89%
7. Artemos-Plus (F&F)	23.15	22.85	23.31	23.10	0.23	22.67	102%
8. Malar-2 Forte (Sadasko)	24.03	23.78	25.57	24.46	0.97	22.67	108%
9. Lonart Forte (Gladon)	22.82	22.66	22.56	22.68	0.13	22.67	100%
10. Artemos-Plus (Tropic)	22.92	23.21	22.40	22.84	0.41	22.67	101%
11. Lonart DS (Richcord)	22.34	21.48	22.62	22.14	0.59	22.67	98%
12. Lonart DS (Gladon)	22.03	22.20	22.04	22.09	0.10	22.67	97%
13. Lonart* (K. Somuah & Sons)	17.26	16.20	18.27	17.24	1.03	22.67	76%
14. Lumartem* (Sarkuff)	22.76	22.94	21.89	22.53	0.56	22.67	99%
15. Lonart* (Josdav Chemists Ltd.)	20.09	18.72	17.65	18.82	1.23	22.67	83%

* Dry powder formula
