# Supplementary material for: Relationship between treatment-seeking behaviour and artemisinin drug quality in Ghana
Source: Malar J. 2012 Apr 6;11:110. doi: 10.1186/1475-2875-11-110 (PMC3339389; doi:10.1186/1475-2875-11-110)
Supplement: Additional file 8 — Artesunate Estimated Quantity. Raw data of estimated artesunate concentrations. [file 1475-2875-11-110-S8.PDF]

Additional File 8: Artesunate Estimated Quantity

Drug Name (Source)	Trial-1	Trial-2	Trial-3	Average	SD	Expected	Percent
16. Gsunate Plus 25* (John Lawrence Chemists Ltd.)							
17. Camoquin Plus (Sadasko)	11.67	11.51	11.68	11.62	0.09	13.01	89%
18. Camosunate Ped** (Sadasko)							
19. Lever Artesunate (Dove)	10.98	11.22	10.51	10.90	0.36	13.01	84%
20. Gsunate (Bendoz)	11.84	12.19	11.90	11.98	0.19	13.01	92%
21. Lever Artesunate (GA Boateng)	11.63	11.50	12.23	11.79	0.39	13.01	91%
22. Arsuamoon (John Lawrence Chemists Ltd.)	12.39	12.24	12.77	12.46	0.27	13.01	96%
23. Co-Artesun (K. Somuah & Sons)	11.49	11.76	11.49	11.58	0.16	13.01	89%
24. Gsunate 100 Kit (Adler)	12.62	11.90	12.00	12.17	0.39	13.01	94%
25. Gsunate 100 Kit (Dove)	12.93	12.71	12.24	12.63	0.36	13.01	97%
26. Coarsucam (Tropic)	11.45	11.64	11.46	11.52	0.11	13.01	89%
27. Malasate 200 (Sadasko)	12.33	12.08	12.85	12.42	0.39	13.01	96%

* Suppository not optimized for our extraction method ** Satchels were opened prior to testing so no analysis was generated
