# Supplementary material for: Relationship between treatment-seeking behaviour and artemisinin drug quality in Ghana
Source: Malar J. 2012 Apr 6;11:110. doi: 10.1186/1475-2875-11-110 (PMC3339389; doi:10.1186/1475-2875-11-110)
Supplement: Additional file 9 — Amodiaquine Estimated Quantity. Raw data of estimated amodiaquine concentrations. [file 1475-2875-11-110-S9.PDF]

Additional File 9: Amodiaquine Estimated Quantity

Drug Name (Source)	Trial-1	Trial-2	Trial-3	Average	SD	Expected	Percent
16. Gsunate Plus 25** (John Lawrence Chemists Ltd.)							
17. Camoquin Plus† (Sadasko)							
18. Camosunate Ped‡ (Sadasko)							
19. Lever Artesunate* (Dove)							
20. Gsunate* (Bendoz)							
21. Lever Artesunate* (GA Boateng)							
22. Arsuamoon (John Lawrence Chemists Ltd.)	41.94	40.56	42.77	41.76	1.11	42.16	99%
23. Co-Artesun (K. Somuah & Sons)	40.18	37.00	38.78	38.66	1.59	42.16	92%
24. Gsunate 100 Kit (Adler)	39.85	38.36	39.02	39.08	0.75	42.16	93%
25. Gsunate 100 Kit (Dove)	39.64	38.94	38.98	39.19	0.40	42.16	93%
26. Coarsucam (Tropic)	40.31	39.99	37.35	39.22	1.62	42.16	93%
27. Malasate 200* (Sadasko)							

* artesunate monotherapy drug; † liquid solution and extraction method was not optimized for this and no analysis was possible; ‡ satchel of drug and extraction method not optimized; ** Suppository not optimized for our extraction method.
