# Supplementary material for: Relationship between treatment-seeking behaviour and artemisinin drug quality in Ghana
Source: Malar J. 2012 Apr 6;11:110. doi: 10.1186/1475-2875-11-110 (PMC3339389; doi:10.1186/1475-2875-11-110)
Supplement: Additional file 10 — Dihydroartemisinin Estimated Quantity. Raw data of estimated dihydroartemisinin concentrations. [file 1475-2875-11-110-S10.PDF]

Additional File 10: Dihydroartemisinin Estimated Quantity

Drug Name (Source)	Trial-1	Trial-2	Trial-3	Average	SD	Expected	Percent	Pre-dilution percent‡
28. Artemos-40 (Tropic)	5.33	6.27	4.60	5.40	0.84	8.79	61%	63%
29. P-Alaxin* (Adler)	7.42	7.96	7.91	7.76	0.30	8.79	88%	83%
30. Alaxin (K. Somuah & Sons)	7.61	8.46	8.77	8.28	0.60	8.79	94%	86%
31. Alaxin (Tropic)	7.73	9.26	8.11	8.36	0.80	8.79	95%	81%
32. Alaxin (GA Boateng)	7.29	8.69	8.46	8.14	0.75	8.79	93%	82%
33. P-Alaxin† (Aseda)	0.50	0.58	0.56	0.55	0.04	0.99	56%	46%
Euratesim (Sigma-Tau)	***8.92***	***8.51***	***9.14***	***8.86***	***0.32***	***8.79***	***101%***	***101%***

* Expired; † Powder form, expected molarity is based on estimate of amount of drug in ~2g; ‡ Pre-dilution percentage kept to demonstrate that estimates of drug concentration are contingent upon correct dilution, and because IC-50s were performed with these concentrations.
