# Supplementary material for: Relationship between treatment-seeking behaviour and artemisinin drug quality in Ghana
Source: Malar J. 2012 Apr 6;11:110. doi: 10.1186/1475-2875-11-110 (PMC3339389; doi:10.1186/1475-2875-11-110)
Supplement: Additional file 11 — IC50 vs. Observed Drug Quantity. Plot of estimated IC50 values vs observed drug quantity. [file 1475-2875-11-110-S11.PDF]

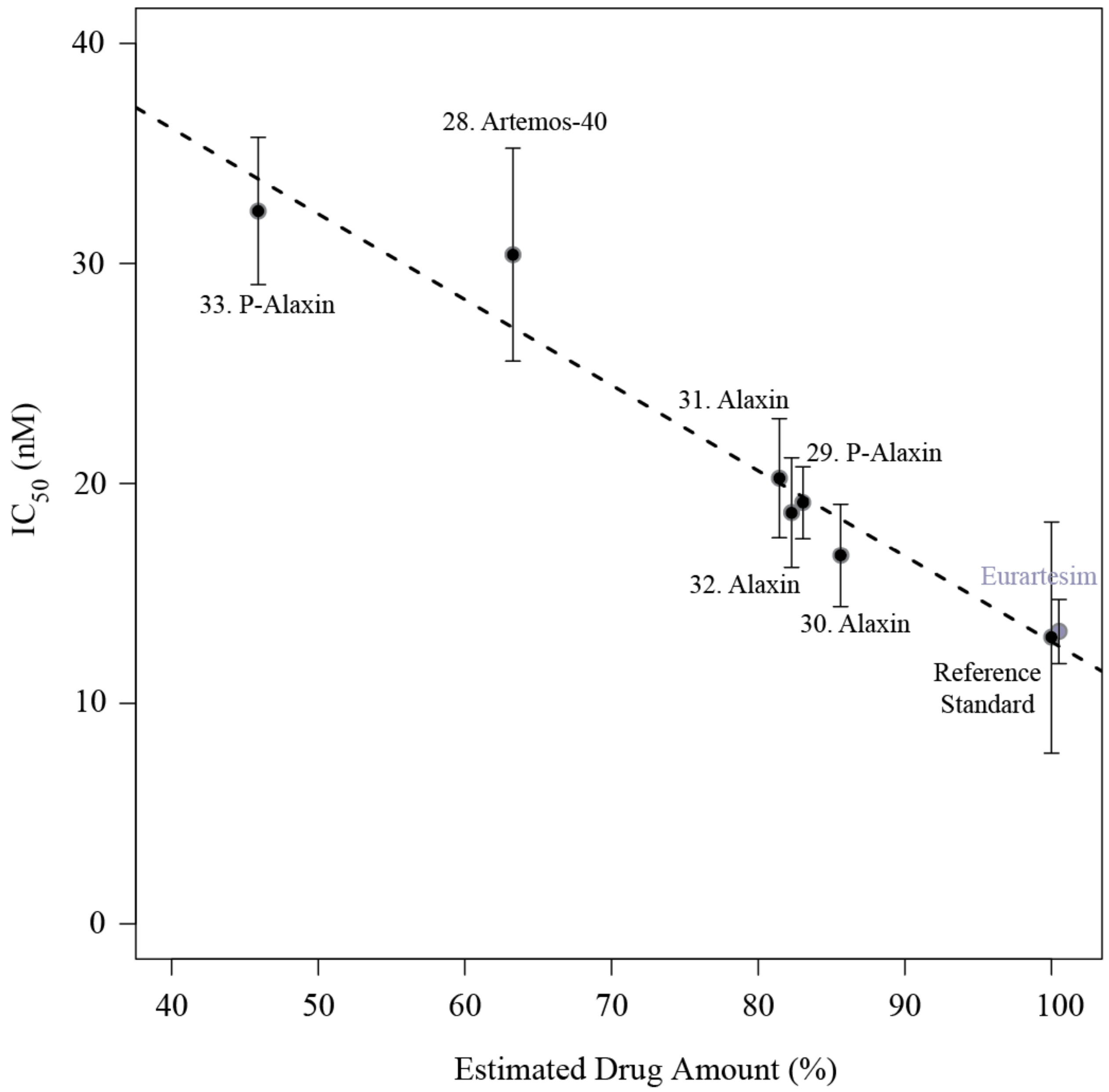

Additional File 11: IC50 vs. Observed drug Quantity

IC50 values and drug quantities reported here are prior to a two-fold dilution. While the dilution series indicated that these drugs are solubility limited, this assay shows a high correlation between NMR-observed concentrations and biological activity.
